# The Effect of State Gratitude on Cognitive Flexibility: A Within-Subject Experimental Approach

**DOI:** 10.3390/brainsci10070413

**Published:** 2020-07-01

**Authors:** Andree Hartanto, Nadia C. H. Ong, Wee Qin Ng, Nadyanna M. Majeed

**Affiliations:** School of Social Sciences, Singapore Management University, 90 Stamford Road, Singapore 178903, Singapore; nadia.ong.2016@socsc.smu.edu.sg (N.C.H.O.); wq.ng.2019@phdps.smu.edu.sg (W.Q.N.); nadyannam.2017@socsc.smu.edu.sg (N.M.M.)

**Keywords:** cognitive flexibility, gratitude, prefrontal cortex, positive emotions

## Abstract

Considerable research has examined the relationship between positive emotion and cognitive flexibility. Less is known, however, about the causal relationship between discrete positive emotions, specifically gratitude, and cognitive flexibility. Given that different positive emotions may dissimilarly affect cognitive functioning, we sought to examine the effect of state gratitude on cognitive flexibility. A pilot study with ninety-five participants was employed to ensure the effectiveness of our gratitude manipulation. One hundred and thirteen participants were recruited for the main study, which utilized a within-subject experimental approach. After the manipulation, participants completed a well-established task-switching paradigm, which was used to measure cognitive flexibility. Contrary to our hypotheses, we did not find any evidence that state gratitude may enhance cognitive flexibility. The current study identified some boundary conditions around the potential benefits of the experience of gratitude.

## 1. Introduction

Past research in the affect literature has demonstrated the significant influence of positive emotion on cognitive flexibility, the ability to shift attention among relevant ideas and to effectively adjust behavior in accordance with changing environmental demands [1,2,3]. For instance, researchers found that individuals induced with positive affect demonstrated increased cognitive flexibility in the global–local shape task [1] and in the dimensional change card sort task [3] compared to those in the neutral condition. Notwithstanding consistent findings evidencing the benefits of positive emotion on cognitive flexibility, most of the past research has largely examined positive emotion as a single construct of positive affect, such as happiness or enjoyment [1,4,5]. However, it is important to note that positive emotion is not a unitary construct [6,7,8]. Rather, research has suggested that different discrete positive emotions may give rise to differential effects on cognitive functioning [9]. One discrete positive emotion that has received less empirical attention in relation to cognition is gratitude. Little is known about the cognitive implications of gratitude as a unique discrete emotion [10]. In light of the scant research in this area, the current study aims to extend the literature by investigating the effect of state gratitude on cognitive flexibility.

Gratitude has been defined as a positive emotion resulting from the positive recognition of benefits received from another person [11,12,13]. The recipient understands that help received from the benefactor is voluntary and without conditions for repayment, thereby prompting the recipient to return the kind gesture as a signal of gratitude, and not merely as an act of exchange to offset what is owed [14]. A universally-esteemed value, the concept of gratitude is heavily embedded in different religions and philosophical ideas as a key component of living a spiritual and virtuous life [15]. Beyond its role as a significant value in life, gratitude as a discrete positive emotion has intrigued many researchers due to its complexity and uniqueness compared to other discrete positive emotions. For instance, unlike other positive emotions, gratitude is categorized by a lack of nonverbal cues, such as the absence of expressive visual display [16] and nonverbal vocal expressions [17]. Furthermore, the experience of gratitude is inherently relational; it extends beyond the individual experiencing it, and towards the social world we live in [18]. Importantly, research on gratitude has demonstrated its positive benefits for physical health [19,20,21], emotional well-being [22,23], and social outcomes [24,25]. Despite the numerous benefits that gratitude confers across these different domains, less has been studied about the impact of gratitude on cognition, specifically in the domain of cognitive flexibility [26]. Therefore, more research is warranted to shed light on the causal effect of state gratitude on cognitive flexibility.

There are several possible mechanisms underlying the effect of state gratitude on cognitive flexibility. First, drawing on the broaden-and-build theory of positive emotions [27], gratitude as a positive emotion can plausibly broaden the scopes of attention and cognition by expanding an individual’s focus to process a wide range of information. In turn, this enhances the range of probable thoughts and actions beyond what the individual was initially capable of and increases access to novel ideas and thoughts. On a neurological level, the broadening effect may be associated with the release of mesolimbic dopamine into brain regions in the prefrontal cortex [28,29], a brain area related to cognitive flexibility [30,31]. Therefore, cognitive flexibility is likely to be implicated and enhanced when one experiences a state of gratitude.

Second, gratitude may enhance cognitive flexibility via its adaptive function as a motivator of self-improvement [32,33]. According to the gratitude and self-improvement model [32], gratitude is an active emotion that motivates and energizes individuals to exercise effort in executing a range of positive self-improvement behaviors. Supporting this model, recent studies have demonstrated that state gratitude may facilitate positive effortful behavior, such as engaging in healthy eating behaviors [34] and physical exercise [35]. Such self-improvement behaviors require individuals to utilize critical executive functions, such as inhibitory control, working memory, and cognitive flexibility. These core executive functions enable individuals to inhibit undesirable behaviors and update their working memory with goal-relevant behaviors, while adjusting and changing their behaviors in a meaningful manner, respectively [36,37]. These findings suggest that the motivating and energizing properties of gratitude may also enhance cognitive flexibility via the self-improvement mechanism.

Third, neuroimaging evidence points to the activation of brain areas related to cognitive flexibility when one experiences gratitude. Neuroimaging studies have linked state gratitude to the activation of brain areas that implicate higher order cognitive functions, including cognitive flexibility. For instance, Fox et al. [38] found that state gratitude is associated with brain activity in the anterior cingulate cortex and medial prefrontal cortex. Similarly, using the “pay it forward” paradigm to manipulate gratitude, Kini et al. [39] found that participants in the gratitude condition displayed significantly greater neural modulation in the medial prefrontal cortex. Given that the medial prefrontal cortex and anterior cingulate cortex have been consistently reported to be involved in cognitive flexibility [40,41,42,43], these findings from neuroimaging studies hint at a possible link between state gratitude and cognitive flexibility.

In view of the potential causal relationship between gratitude and cognitive flexibility, the current study aims to examine the effects of state gratitude on cognitive flexibility. A within-subject experimental approach was employed to demonstrate potential causal evidence of the effect of gratitude on cognitive flexibility. The within-subject experimental approach is desirable, given that it increases statistical power and minimizes error rates due to individual differences [44]. To prevent an order effect as a potential confounder, counterbalancing was utilized.

The present study measured cognitive flexibility using a well-established task-switching paradigm, which yields two performance indicators: switch costs and mixing costs [45]. Switch costs represent performance costs that arise when switching from one task set to another, reflecting local transient control processes [46,47]. In contrast, mixing costs reflect performance costs incurred when having to monitor two competing task-sets during task-switching, as opposed to performing a single task-set, reflecting global sustained control mechanisms [48,49]. Measuring both switch and mixing costs would allow us to capture two important distinct control mechanisms of cognitive flexibility and specify how gratitude may relate to these two mechanisms. 

Taking into account neuroimaging findings on gratitude, along with the broaden-and-build theory of positive emotions [27] and the self-improvement model of gratitude [32], we hypothesized that participants induced with a grateful state would have enhanced cognitive flexibility, evidenced by lower switch costs and mixing costs, as compared to participants induced with a neutral state.

## 2. Pilot Study

Prior to the main experiment, a pilot study was conducted to ensure the effectiveness of our gratitude manipulation. Based on the suggestion by Hauser et al. [50] we chose to conduct a pilot study to avoid any manipulation checks in the main study. This was to ensure that we could minimize potential demand characteristics due to the within-subject experimental approach that was employed in the main study. State gratitude was induced by adapting a well-established manipulation protocol from Emmons and McCollough [51]. Participants in the gratitude condition were instructed to list five events they felt grateful for over the past week, whereas participants in the control condition listed five events that occurred over the past week.

### 2.1. Materials and Methods

#### 2.1.1. Participants

Ninety-five volunteers and undergraduates (female = 61) from a local university in Singapore participated in the pilot study for extra course credit. The average age of the participants was 22.62 years (*SD* = 5.79). As the current study employed a within-subject design, participants were required to complete two separate sessions with a seven-day interval between each session. For each participant, one session included the gratitude manipulation (gratitude condition) whereas the other session consisted of the neutral manipulation (control condition). The time interval in the pilot study was designed to mimic the interval in the main study. The order of the sessions was counterbalanced across participants.

#### 2.1.2. State Gratitude Scale

The State Gratitude Scale (SGS) was used as a manipulation check to measure state gratitude. Participants rated their agreement with five items concerning how they felt at the present moment using a five-point Likert scale (1 = strongly disagree; 5 = strongly agree). The scale has acceptable reliability (*α* = 0.88). 

#### 2.1.3. International Positive and Negative Affect Schedule Short Form (I-PANAS-SF)

I-PANAS-SF was used to measure positive and negative affect [52]. Participants rated how strongly they agreed or disagreed with ten items measuring how they felt at the current moment on a five-point Likert scale (1 = strongly disagree; 5 = strongly agree). The scale has acceptable reliability for both positive affect (*α* = 0.80) and negative affect (*α* = 0.85). An additional eleventh item, “blessed”, was included as a measure of gratitude and served as a manipulation check item.

#### 2.1.4. Procedure

The pilot study consisted of two separate sessions conducted seven days apart. In each session, participants were randomly assigned to either the gratitude or control condition, such that they were assigned to both conditions after completing the pilot study. Participants were instructed to complete a writing task that served to induce gratitude (gratitude condition) or a neutral state (control condition). In the gratitude condition, participants were presented with the following instructions: “There are many things in our lives, both large and small, that we might be grateful about. Think back over the past week and write down on the lines below up to five things in your life that you are grateful or thankful for.” In the neutral (control) condition, participants were given the following instructions: “What are some of the routine daily events that occurred to you in the past week? Think back over the past week and list in the space below five routine events that you encountered. (routine events include making breakfast, running errands, brushing your teeth etc.).” Participants were required to complete the SGS, I-PANAS-SF, and a short demographic questionnaire at the end of each session.

### 2.2. Results

To examine if our gratitude manipulation was successful, a paired-sample *t*-test was conducted on each item of both the SGS and the I-PANAS-SF. Supporting the effectiveness of our manipulation in inducing state gratitude, participants reported higher levels of gratitude in the gratitude condition as compared to the control condition for all five items in the state gratitude scale, “I feel grateful”, “I feel a warm sense of appreciation”, “I am happy to have been helped by others”, “I have benefitted from the goodwill of others”, and “I have been treated with generosity”. More importantly, there was no significant difference between the gratitude condition and control condition in all ten items in the I-PANAS-SF, except for “blessed”. These findings, presented in Table 1, demonstrate that experimentally manipulating gratitude enhanced feelings of gratitude but did not induce other positive and negative emotions, supporting the effectiveness and specificity of our manipulation.

## 3. Main Study

Having established the effectiveness and specificity of our gratitude manipulation, we conducted our main study to examine the effect of state gratitude on cognitive flexibility. In addition to the gratitude manipulation, participants were required to complete a task-switching paradigm that measured cognitive flexibility. Similar to the pilot study, participants completed two separate sessions with a seven-day interval between each session. The order of the sessions was counterbalanced.

### 3.1. Materials and Methods

#### 3.1.1. Participants

One hundred and thirteen undergraduates (female = 87) from a local university in Singapore who did not participate in the pilot study were recruited in exchange for one course credit or monetary compensation of S$5. The average age of the participants was 21.88 years (*SD* = 1.67), and the ages ranged from 19 to 27 years. The study was approved by SMU IRB on 16 December 2019; approval number IRB-19-123-A100(1219). We have also submitted an IRB modification which was approved on 17 January 2020; approval number IRB-19-123-A100-M1(120).

#### 3.1.2. Magnitude–Parity Switching Task

The magnitude–parity switching task, a well-established task-switching paradigm, was employed to measure cognitive flexibility [45,53]. In the magnitude task, participants were asked to categorize a target digit as quickly and as accurately as possible according to its magnitude (whether the target digit was greater or smaller than the number “5”). In the parity task, participants had to categorize a target digit according to its parity (whether target digit was odd or even). An image of a row of big blue circles and small yellow circles cued the magnitude task. In contrast, the parity task was cued by an image of two rows of shapes: a top row of odd-numbered blue squares and a bottom row of even-numbered yellow squares. To respond to the different cues, participants used their index fingers to press a key (“d”) labeled “3” to stipulate an odd number or magnitude smaller than five and a key (“k”) labeled “6” to stipulate an even number or magnitude greater than five.

There are three types of trials in the current task-switching paradigm [54]. They include switch trials (trials in which one must switch from completing one task to a different task, e.g., a magnitude task following a parity task), repeat trials (trials that are of the same task as the previous trial, e.g., a magnitude task followed by another magnitude task), and pure trials (trials in which there is only a single task set, e.g., magnitude tasks only). Mixed blocks consist of both switch and repeat trials, whereas pure blocks consist of only pure trials. Each participant completed two practice blocks (a magnitude block and a parity block with eight trials each), followed by two pure blocks (pure magnitude block and pure parity block of 20 trials each) and three mixed blocks (20 switch trials and 20 repeat trials each). There were a total of 60 switch trials, 60 repeat trials, and 40 single-task trials (20 pure parity trials and 20 pure magnitude trials).

At the beginning of each trial, a fixation point appeared for 350 ms. This was followed by a blank screen for 150 ms and a subsequent cue that appeared above the fixation point and remained on the screen until the end of each trial. After fixating for 250 ms, the target digit appeared in the center of the screen and remained until the participant’s response was entered. Thereafter, an intertrial interval of 850 ms preceded the onset of the next trial.

Cognitive flexibility was indexed by switch costs and mixing costs. Switch costs refer to the performance difference between switch trials and repeat trials, while mixing costs refer to the performance difference between repeat trials and pure trials. In the current study, switch costs and mixing costs were calculated using accuracy and reaction time data, and the combination of accuracy and reaction time obtained via binning scores [55].

#### 3.1.3. Procedure

The main study was conducted in a psychology lab where participants completed the study in individual cubicles. Participants were randomly assigned to either the gratitude or control condition in the first session. They completed the writing task relevant to each condition in the respective session, followed by the magnitude–parity task-switching paradigm. The magnitude–parity task-switching paradigm was implemented using E-Prime version 3.0 software (Psychology Software Tools, Pittsburgh, PA) [56]. Similar to the pilot study, participants completed both sessions with a seven-day interval between the sessions. 

### 3.2. Results

#### 3.2.1. Accuracy

The mean accuracy rates for each condition are shown in Table 2. For the accuracy of switch costs, a two-factor within-subject ANOVA was performed with Gratitude (experimental condition vs. control condition) as the first within-subject factor and Switching (switch trials vs. repeat trials) as the second within-subject factor. We found a significant main effect of Switching, *F*(1, 112) = 94.39, *p* < 0.001, *η_p_*^2^ = 0.46, suggesting that the accuracy rates were significantly lower in switch trials compared to repeat trials. However, there was no main effect of Gratitude, *F*(1, 112) = 0.01, *p* = 0.909, *η_p_*^2^ = 0.00, and the interaction between Switching and Gratitude was not significant, *F*(1, 112) = 0.28, *p* = 0.598, *η_p_*^2^ = 0.002, suggesting that gratitude did not affect switch costs in accuracy.

We also conducted a two-factor within-subject ANOVA for the accuracy of mixing costs with Gratitude (experimental condition vs. control condition) as the first within-subject factor and Mixing (repeat trials vs. pure trials) as the second within-subject factor. Similarly, we found a significant main effect of Mixing, *F*(1, 112) = 6.51, *p* = 0.012, *η_p_*^2^ = 0.06, suggesting that the accuracy rates were significantly lower in repeat trials compared to pure trials. However, we did not find a significant main effect of Gratitude *F*(1, 112) = 0.33, *p* = 0.566, *η_p_*^2^ = 0.003. There was also no significant interaction effect between Mixing and Gratitude *F*(1, 112) = 0.85, *p* = 0.359, *η_p_*^2^ = 0.01, suggesting that the experience of gratitude did not affect participants′ accuracy of mixing costs.

#### 3.2.2. Reaction Time

Prior to our data analyses, reaction times that were either below 200 ms or 2.5 standard deviations above or below each participant’s mean were excluded for both pure and mixed blocks separately. Only trials with accurate responses were analyzed for reaction time. The mean reaction times for each condition are shown in Table 2. After the outlier trials were removed, a two-factor within-subject ANOVA was performed for the reaction time of switch costs with Gratitude (experimental condition vs. control condition) as the first within-subject factor and Switching (switch trials vs. repeat trials) as the second within-subject factor. As expected, we found a significant main effect of Switching, *F*(1, 112) = 110.01, *p* < 0.001, *η_p_*^2^ = 0.50, suggesting that participants’ reaction times were significantly slower in switch trials than repeat trials. We also found a significant main effect of Gratitude, *F*(1, 112) = 6.57, *p* = 0.012, *η_p_*^2^ = 0.06. However, we did not find a significant interaction between Switching and Gratitude *F*(1, 112) = 1.06, *p* = 0.306, *η_p_*^2^ = 0.01, suggesting that switch costs measured by reaction times were not affected by gratitude.

With regard to the reaction time of mixing costs, a similar two-factor within-subject ANOVA was performed with Gratitude (experimental condition vs. control condition) as the first within-subject factor and Mixing (repeat trials vs. pure trials) as the second within-subject factor. We found a significant main effect of Mixing *F*(1, 112) = 214.31, *p* < 0.001, *η_p_*^2^ = 0.66, suggesting that participants’ reaction times were significantly slower for repeat trials than for pure trials. We also found a significant main effect of Gratitude *F*(1, 112) = 5.51, *p* = 0.021, *η_p_*^2^ = 0.05, and a significant interaction effect between Mixing and Gratitude, *F*(1, 112) = 5.41, *p* = 0.022, *η_p_*^2^ = 0.05, where participants in the gratitude condition had higher mixing costs than those in the control condition. However, it is noteworthy that the results were not significant after adjusting for multiple comparisons.

#### 3.2.3. Combining Accuracy and Reaction Time

Lastly, we also combined participants’ accuracy and reaction times to calculate switch costs and mixing costs using a binning procedure [55,57]. This method has been shown to yield higher construct validity and reliability than pure reaction time and accuracy scores. The binning procedure involved several steps. First, for each participant, reaction times (RTs) below 200 ms or 2.5 standard deviations away from the individual’s mean RT were excluded. Subsequently, we calculated the mean RT of accurate repeat trials for each participant and subtracted this value from the RT of each accurate switch trial. Because this RT difference was calculated for each accurate switch trial for each participant, we referred to these as trial-based switch costs in switching tasks. Next, all participants’ trial-based switch costs were combined, rank ordered into deciles, and assigned a bin value ranging from 1 to 10. Greater bin values were assigned to trials with relatively larger switch costs (i.e., poorer switching efficiency), whereas smaller bin values were assigned to trials with relatively smaller switch costs (i.e., better switching efficiency). Importantly, a penalty was imposed by assigning a higher bin value of 20 to all incorrect responses [57]. Lastly, we averaged all of the bin values each participant received for each trial to create a single index of switch costs in terms of bin scores, which ranged from 1 to 20. Overall, lower bin scores for task switching reflect better task-switching performance.

A paired-sample *t*-test was conducted to compare switch costs in binning scores between the gratitude condition and control condition. We found no significant difference in the switch costs between the gratitude condition (*M* = 7.16, *SD* = 1.71) and control condition (*M* = 7.14, *SD* = 1.67), *t*(112) = 0.24, *p* = 0.810. We also conducted a paired-sample *t*-test to compare mixing costs in binning scores between the gratitude condition and control condition. Similarly, we did not find significant difference in the mixing costs between the gratitude (*M* = 6.43, *SD* = 1.55) and control (*M* = 6.37, *SD* = 1.52) conditions, *t*(112) = 1.01, *p* = 0.313. These results suggest that being in a state of gratitude did not affect switch costs and mixing costs calculated using the binning procedure. 

#### 3.2.4. Bayesian Analyses

In view of the non-significant findings with respect to accuracy and reaction time, we conducted Bayesian two-factor within-subject ANOVAs using JASP version 0.12.2 software (JASP Team, Amsterdam, Netherlands) [58] to further examine the null effect in the aforementioned interactions. Based on the recommendations by Lee and Wagenmakers [59], we estimated the Bayes factor based on comparisons between the models with two main effects (Gratitude and Switching or Gratitude and Mixing) and the model with the respective additional interaction term. As shown in Table 3, our Bayesian analyses indicated anecdotal to moderate evidence in favor of the null hypothesis for both interactions, further providing support for the finding that gratitude did not affect switch costs or mixing costs in accuracy or reaction time. Additionally, Bayesian paired-sample *t*-tests were also conducted to investigate the non-significant finding with respect to binned scores. As shown in Table 3, we found moderate evidence in favor of the null hypothesis.

## 4. Discussion

In the current study, we aimed to examine whether inducing state gratitude would temporarily enhance an individual’s cognitive flexibility. However, contrary to our hypotheses, we did not find any evidence that state gratitude may enhance cognitive flexibility, measured by switch costs and mixing costs in a well-established task-switching paradigm [45]. Using Bayesian analyses, we also found anecdotal to moderate evidence in favor of the null hypothesis.

By employing an experimental approach, our study provides insight on the causal relationship between gratitude and cognitive flexibility. Specifically, the findings of the current study may suggest that the effect of positive emotions may not be the same for each discrete positive emotion; not all positive discrete emotions may enhance cognitive flexibility. This is in line with past evidence that positive discrete emotions may be differently associated with similar cognitive, motivational, and social functions [7,9,60]. For instance, Gable and Harmon-Jones [61] found that positive emotions with high approach motivation reduced the breadth of attention, while positive emotions with low approach motivation increased the breadth of attention. Thus, it is plausible that the cognitive flexibility advantages of positive emotions are limited to discrete positive emotions with lower approach motivation, such as joy [3], but not discrete positive emotions with higher approach motivation, such as gratitude.

Moreover, the current findings could suggest that the practice of gratitude may not always entail positive outcomes [62]. This is consistent with prior findings suggesting that gratitude may impose costs in some situations. For instance, a study conducted by Ng et al. [63] found that participants induced to be in a grateful state were more likely to privately conform to a popular choice despite it being fundamentally incorrect. Another recent study by Ksenofontov and Becker [64] reported that expressing gratitude to high-power groups that had committed an offence resulted in less protest behaviors in low-power groups. Although gratitude has been consistently linked with socioemotional and health benefits [20,23,65], the current study contributes to the literature by demonstrating that the practice of gratitude may not always be advantageous, especially in the domain of cognitive flexibility.

Nevertheless, the current study is not without limitations. First, given that the study only employed a single task-switching paradigm (i.e., magnitude–parity switching task), the null finding might be task-specific and cannot be generalized to other tasks measuring cognitive flexibility. In view of the potential task impurity issue found in cognitive tasks [66], it would be worthwhile for future studies to replicate the current study using other variants of the task-switching paradigm, such as the color–shape switching task [67,68] and the number–letter switching task [69,70]. Second, although the manipulation check in our pilot study supports the effectiveness and specificity of our state gratitude manipulation, it is plausible that the gratitude manipulation was not successful in the main study, which resulted in the null effect. It is also plausible that the gratitude manipulation was short-lived and its effect on cognitive flexibility could be transient. Third, due to the nature of our gratitude manipulation (e.g., unblinded scenario), as well as the use of the within-subject experimental approach, it is plausible that our participants might have been able to guess the hypotheses of the current study, resulting in demand characteristics. Thus, future studies should consider including a manipulation check in the main study and employing a stronger manipulation for state gratitude to ensure that the emotion can last throughout the experiment.

## 5. Conclusions

In summary, we did not find any evidence that state gratitude may enhance cognitive flexibility. The present finding allows us to identify a significant boundary condition around the potential benefits of the experience of gratitude. Despite previous findings consistently demonstrating the association between gratitude and both physical and socioemotional well-being [20,71,72], the present study indicates that the benefits of gratitude may not apply across all domains in life. It may be argued that gratitude does not confer benefits on higher-order cognitive performance, such as cognitive flexibility.

## Figures and Tables

**Table 1:** Manipulation check in gratitude condition and control condition using State Gratitude Scale and International Positive and Negative Affect Schedule Short Form (I-PANAS-SF).

	Gratitude		Control		*t*	*p*
Items	*M*	*SD*	*M*	*SD*		
**State Gratitude Scale**						
I feel grateful	4.14	0.72	3.77	0.92	3.48	0.001
I feel a warm sense of appreciation	4.11	0.61	3.61	0.98	4.88	< 0.001
I am happy to have been helped by others	4.28	0.72	4.01	0.88	2.74	0.007
I have benefitted from the goodwill of others	4.23	0.71	3.92	0.94	3.17	0.002
I have been treated with generosity	4.18	0.73	3.76	0.90	4.12	< 0.001
**I-PANAS-SF**						
Upset	4.16	0.87	4.09	0.92	0.63	0.530
Hostile	4.43	0.71	4.34	0.83	1.35	0.181
Alert	2.95	1.12	2.84	1.13	0.88	0.383
Ashamed	4.31	0.80	4.21	1.02	0.90	0.368
Inspired	3.13	0.97	2.92	1.05	1.83	0.070
Nervous	4.03	1.03	3.95	1.02	1.02	0.312
Determined	3.25	1.02	3.13	1.10	1.20	0.232
Attentive	3.31	0.93	3.22	1.00	0.82	0.412
Afraid	4.11	0.95	4.02	1.00	0.94	0.348
Active	2.97	1.07	2.97	1.05	0.00	1.000
Blessed	4.02	0.87	3.49	1.03	4.47	< 0.001

**Table 2:** Reaction times, accuracy rates, and task-switching costs in magnitude–parity switching task.

Trials and performance indicators	Accuracy ^1^			Reaction Time (RT) ^1^		
	Gratitude ^2^	Control ^2^	*t* ^3^	Gratitude ^2^	Control ^2^	*t* ^3^
Pure	0.95 (0.07)	0.95 (0.07)	1.14	480.21 (89.17)	472.36 (97.67)	0.76
Repeat	0.93 (0.11)	0.94 (0.10)	−0.16	727.15 (220.67)	676.77 (182.47)	2.62 **
Switch	0.89 (0.11)	0.89 (0.11)	0.32	847.95 (289.24)	784.34 (266.43)	2.40 *
Switch Costs	−0.04 (0.06)	−0.05 (0.06)	0.53	120.81 (134.92)	107.57 (133.91)	1.03
Mixing Cost	−0.02 (0.08)	−0.01 (0.08)	−0.92	246.94 (198.90)	204.41 (181.76)	2.33 *

^1^*N* = 113. ^2^ The values presented are the means and standard deviations (*SD*s) of the trials where *SD*s are shown in parentheses. ^3^ * *p* < 0.05, ** *p* < 0.01.

**Table 3:** Summary of interaction effects of Gratitude and Switching/Mixing on accuracy and reaction time using Bayesian analyses.

Dependent Variable	BF_10_	BF_01_	Evidence Category for H_0_
Switch costs (accuracy) ^1,2^	0.15	6.76	Moderate
Switch costs (RT) ^1,2^	0.15	6.90	Moderate
Switch costs (binned) ^1^	0.11	9.35	Moderate
Mixing costs (accuracy) ^1,2^	0.18	5.49	Moderate
Mixing costs (RT) ^1,2^	0.56	1.78	Anecdotal
Mixing costs (binned) ^1^	0.17	5.81	Moderate

^1^*N* = 113. ^2^ The Bayes factor was computed based on the comparison between the model of two main effects and the model with additional interaction terms (i.e., the interaction model), using a non-informative Jeffreys-Zellner-Siow (JZS) default prior. The classification scheme for the interpretation of the Bayes factors was based on Lee and Wagenmakers [59].
